# Western Australian adolescent emotional wellbeing during the COVID-19 pandemic in 2020

**DOI:** 10.1186/s13034-021-00433-y

**Published:** 2022-01-13

**Authors:** H. M. Thomas, K. C. Runions, L. Lester, K. Lombardi, M. Epstein, J. Mandzufas, T. Barrow, S. Ang, A. Leahy, M. Mullane, A. Whelan, J. Coffin, F. Mitrou, S. R. Zubrick, A. C. Bowen, P. W. Gething, D. Cross

**Affiliations:** 1 Telethon Kids Institute, Perth, Australia; 2 University of Western Australia, Perth, Australia; 3 Edith Cowan University, Perth, Australia; 4 Perth Children’s Hospital, Perth, Australia; 5 Curtin University, Perth, Australia

**Keywords:** COVID-19, Adolescent wellbeing, CHU9D

## Abstract

**Background:**

The impacts of the COVID-19 pandemic have been vast and are not limited to physical health. Many adolescents have experienced disruptions to daily life, including changes in their school routine and family’s financial or emotional security, potentially impacting their emotional wellbeing. In low COVID-19 prevalence settings, the impact of isolation has been mitigated for most young people through continued face-to-face schooling, yet there may still be significant impacts on their wellbeing that could be attributed to the pandemic.

**Methods:**

We report on data from 32,849 surveys from Year 7–12 students in 40 schools over two 2020 survey cycles (June/July: 19,240; October: 13,609), drawn from a study of 79 primary and secondary schools across Western Australia, Australia. The Child Health Utility Index (CHU9D) was used to measure difficulties and distress in responding secondary school students only. Using comparable Australian data collected six years prior to the pandemic, the CHU9D was calibrated against the Kessler-10 to establish a reliable threshold for CHU9D-rated distress.

**Results:**

Compared to 14% of responding 12–18-year-olds in 2013/2014, in both 2020 survey cycles almost 40% of secondary students returned a CHU9D score above a threshold indicative of elevated difficulties and distress. Student distress increased significantly between June and October 2020. Female students, those in older Grades, those with few friendships or perceived poor quality friendships, and those with poor connectedness to school were more likely to score above the threshold.

**Conclusions:**

In a large dataset collected during the first year of the COVID-19 pandemic, the proportion of secondary school students with scores indicative of difficulties and distress was substantially higher than a 2013/2014 benchmark, and distress increased as the pandemic progressed, despite the low local prevalence of COVID-19. This may indicate a general decline in social and emotional wellbeing exacerbated by the events of the pandemic.

*Trial registration:* ANZCTRN (ACTRN12620000922976). Retrospectively registered 17/08/2020. https://www.anzctr.org.au/Trial/Registration/TrialReview.aspx?id=380429&isReview=true.

**Supplementary Information:**

The online version contains supplementary material available at 10.1186/s13034-021-00433-y.

## Background

Globally, the SARS-CoV-2 virus (COVID-19) has impacted how communities interact, children are educated, and people live and work [1]. Within the first three months of the COVID-19 pandemic, approximately half of the global school student population experienced some level of school closures [2]. In some countries, learning from home became the new norm for 2020, with significant commentary on the potential psychosocial impacts of this for young people, including the consequences of decreased social opportunities and potential for isolation and loneliness during long periods of time at home [3–5]. Data from countries with high local transmission, such as Italy [6], the Netherlands [7] and Germany [8], show declines in quality of life for children and adolescents.

However, in high-transmission contexts, it is unclear whether this impact on quality of life is due to the limitations imposed by lockdown, or by more existential concerns. We examined wellbeing among adolescents in Western Australia in 2020, where low COVID-19 transmission and little intensive lockdown were experienced. As in other parts of the world with low COVID-19 prevalence, school closures in Western Australia were relatively brief (less than two weeks), and students spent the majority of 2020 attending school in person. Although impacts such as isolation were thus unlikely for young Western Australians, they may nonetheless have observed the broader impact of COVID-19 on others in the world, and experienced disruptions including altered parental work arrangements, less household income, increased uncertainty, and impacts on the mental health of adults in their lives [9, 10]. These factors have the potential to impact the psychosocial wellbeing of young people irrespective of local disease prevalence. A study of Australian adolescents using a convenience sample provided descriptive data on the perceived impact of COVID-19 on their mental health [11], but it is unclear if these findings reflect a deterioration of wellbeing against established pre-pandemic benchmarks. The current study provides a test of this possibility with a large sample of young people in Western Australia.

**Fig. 1:**
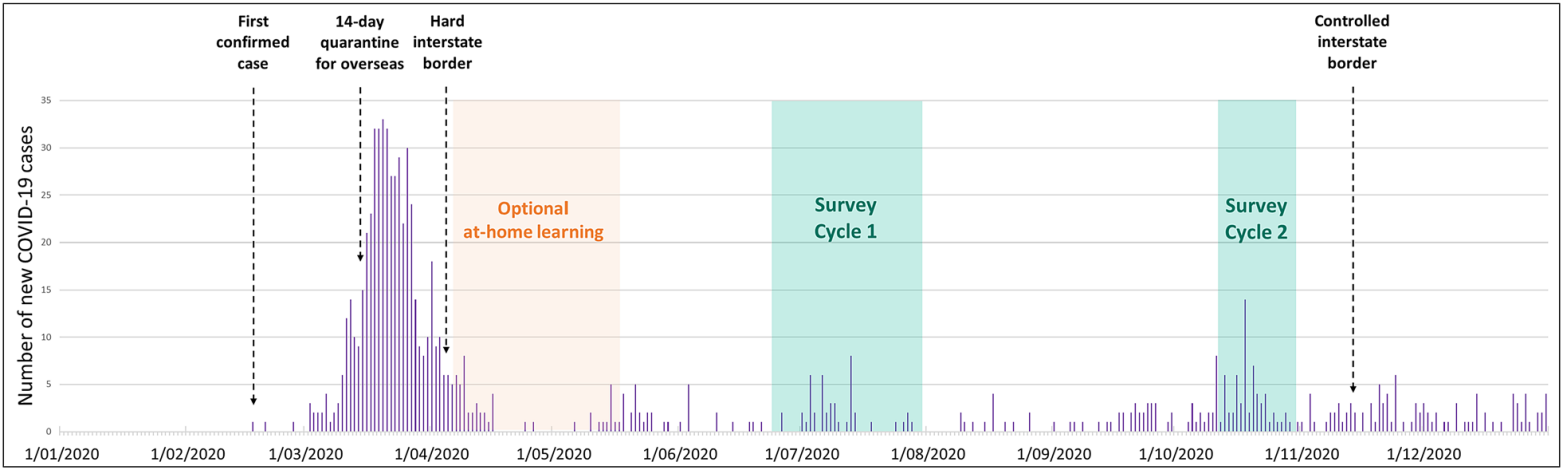
Study context: COVID-19 cases reported in Western Australia during 2020. Orange shade indicates period of optional at-home learning for Western Australian students; green shade indicates periods of survey collection for Cycles 1 and 2

The first case of COVID-19 was recorded in Western Australia on February 21, 2020 (Fig. 1). In early March 2020, a 14 day quarantine was established for all overseas arrivals and by March 22, 2020, a ‘hard’ interstate border was introduced which remained in place until November 2020. New school learning arrangements for students were implemented on March 26, 2020, with families encouraged to keep their children learning from home. Cases of community-acquired COVID-19 dramatically declined to zero by mid-April. Schools re-opened for Term 2 on April 29, 2020 and on May 14, 2020, the Western Australian government announced all children must return to school by the week beginning May 18, 2020.

The first data collection (C1) for the current (“DETECT”) study was in June 2020, when all primary and secondary students were back at school and the State’s COVID-19 case load was reduced to few or no new cases/day, all of which were recorded in quarantined overseas travellers. Western Australia remained a region of low COVID-19 prevalence, with no local transmission after April 13, 2020, and a total of 979 cases and 9 deaths recorded throughout 2020. A second cross-sectional data collection (C2) was conducted in October 2020, after a sustained period free of COVID-19 community transmission, and progression of the state through the phases of a recovery plan. During this time there was continued high media coverage of the impact of the virus globally, including a 112 day lockdown in Melbourne located in Victoria, Australia.

Early in the pandemic, studies from other countries indicated that school closures and other pandemic events could be significantly impacting the wellbeing of children [12]. We aimed to determine whether, in a setting such as Western Australia where the impact of the pandemic in 2020 was limited compared to other global jurisdictions, there is evidence of possible significant psychological impact of COVID-19 in the face of very limited local threat. Although our cross-sectional data do not permit attributing causality to the impact of the pandemic, we have tested the hypothesis that even in the absence of high rates of COVID-19 locally, the wellbeing of adolescents in Western Australia would be lower than that noted at a benchmark assessment conducted in 2013/2014. Furthermore, by collecting two cycles of data during 2020, we have examined whether wellbeing shifted during the course of the pandemic.

We were also concerned to examine potential inequity in the wellbeing of young people. In Australia, Aboriginal and Torres Strait Islander young people have been found to be at elevated risk of emotional distress and poor mental health outcomes [13–15]. Rates of depression and other mental disorders amongst children and adolescents have been found to be elevated in rural and regional areas of Australia [16], in particular in outer regional areas [17]. We also examined whether students whose families spoke a language other than English at home were at differential risk for poor wellbeing than other students, as has been found for Australian parents during the COVID-19 pandemic [18].

Finally, we examined several potential risk and protective factors for wellbeing, including the peer context and the school context. Based on prior research (e.g., [19–21], we hypothesized that students with a greater number of friends and those who reported greater quality of friendships would report greater wellbeing, and students who felt more connected to their school would also report greater wellbeing.

## Methods

### Setting

On May 1, 2020, the Western Australian government partnered with the Telethon Kids Institute to conduct the DETECT Schools study, to screen for COVID-19 via swab testing (conducted in 40 primary and secondary schools) and measure wellbeing in school communities (conducted in 79 schools). Schools were nominated by the Department of Education for participation, and selected to include representation across regions, settings, primary and secondary schools whilst balancing the operational needs of the study. In May 2020, two months after the first COVID-19 case in Western Australia, this prospective observational cohort surveillance study was launched across the State. For full details of the study, see the published protocol [22].

### Procedure

Although active consent was required for adult survey completion, passive consent was approved for the collection of two cycles of student wellbeing survey data to enable the engagement of a large and representative sample. Parents of students were given the opportunity to opt their children out of the study: students not opted out were offered the survey online or in hard copy at school. Students provided assent prior to completing the survey. The survey did not contain any mandatory fields, and students could complete as much or as little of the survey as they liked.

Wellbeing surveys were administered to Grades 4 to 12 (10–18 years of age) students, their parents and teachers. Parents of Kindergarten to Grade 3 (4–9 years of age) students provided data about their children’s wellbeing. This paper addresses the two cycles of data collected from students in Grades 7 to 12 (12–18 years of age) only, in which the key outcome measure (Child Health Utility Index (CHU9D)) was surveyed. While included in the wider study, primary school students were not asked the questions of the CHU9D. The findings from primary school student surveys are reported seperately.

Surveys were conducted during school hours in two cross-sectional cycles separated by four months at each participating school. Most surveys were completed online using the REDCap platform [23]. Some students required hard copies of the survey to complete. Each school had a coordinator equipped to administer the survey, and mental health support information was made available for all students in the schools completing the survey.

### Measures

#### Demographics

Demographic information collected from each student included school Grade level, gender, language other than English spoken at home (LOTE) status, Aboriginal and/or Torres Strait Islander background, whether they resided at a residential college, the educational region within Western Australia (North and South Metropolitan, Goldfields, Kimberley, Midwest, Pilbara, Southwest, and the Wheatbelt), and the number of residents in their household.

#### Wellbeing

The wellbeing survey asked participants to respond to the nine items of the Child Health Utility Index (CHU9D), which was designed [24, 25] and has been previously used in an Australian adolescent cohort [26] as a preference-based health-related quality-of-life tool, but was used in this study as an index of difficulties and distress. Only students in Grades 7–12 were asked to complete the questions from the CHU9D scale, which comprises nine questions about a child’s experiences “today” in relation to feeling worried, sad, pain, tired, annoyed, or experiencing difficulties with schoolwork, sleep, daily routine, and activities. Each item comprises a 5-category Likert rating, and responses are progressively scaled for severity from no (1) or less severe outcomes to more or most (5) severe outcomes. A factor analysis of the CHU9D items yielded one factor (C1 α = 0.85). The CHU9D items were summed to create a total score with a higher score representing greater difficulties and distress (range 9–45).

The most recent collection of these data in a comparable pool of Australian adolescents was in 2013/2014 [27], when a cohort of young people responded to both the CHU9D and the Kessler-10 (K10; [28]), a well-validated measure of mental health distress. Using these data, we established a reliable threshold for CHU9D-rated difficulties and distress by calibrating CHU9D scores from Grade-level-matched respondents against their scores for the K10. Analysis of the distributions for both the CHU9D and K10, along with Receiver Operator Curve Analysis [29], revealed excellent correspondence between these two measures. Based on these data, a score of ≥ 20 on the CHU9D was established as the threshold representing elevated (moderate to high) difficulties and distress.^1^ The cohort of students scoring above this threshold can be stratified into those reporting moderate (CHU9D score between 20 and 25) and high (CHU9D > 25) levels of difficulties and distress. For analyses, CHU9D scores above the threshold of ≥ 20 have been used to represent elevated (moderate through high) difficulties and distress. To assess the hypothesis that adolescent wellbeing during 2020 was poorer than that measured before the pandemic, we compared the proportion of respondents scoring above this threshold between 2013/2014 and 2020.

#### Social context

Measures of the students’ social context were collected, to examine potential protective factors for distress. Friendship measures included two individual items describing the number and quality of friends. Students were asked ‘About how many friends do you have who you either hang out with, talk to on the phone, regularly send messages to, either through social media, chat, gaming, or other ways online, or get together with socially?’ with response options ranging from zero to 20 + friends. Students were also asked ‘How much can you rely on your friends for help if you have a serious problem?’ with response options ‘a lot’, ‘some’, ‘a little’ and ‘not at all’.

Students’ sense of connectedness to school was measured using items adapted from the ACER School Life Questionnaire. School connectedness items were measured on a 5-point scale from ‘strongly disagree’ to ‘strongly agree’, with respondents asked to indicate how much their school was a place where they felt happy; liked to go each day; found learning fun; felt safe and secure; liked learning and gained enjoyment from attending.

All survey items can be found in the appendix of the published protocol [22].

### Data analysis

Chi-square analysis in SPSS was used to determine differences in demographic and survey responses for categorical item data. Generalised linear mixed modelling with a negative binomial link function was used in SPSS to examine mean differences in CHU9D scores at C1 and C2, accounting for school-level clustering. To better understand who might be most vulnerable to high levels of difficulties and distress during COVID-19, STATA was used for multi-level regression models that considered individual-level predictors, including participants age, gender, LOTE status, Aboriginal and Torres Strait Islander status, region within Western Australia, number of people living in the child’s home, child’s number and quality of friends and child’s connectedness to school. To simplify reporting, these analyses examined moderate and high levels of difficulties and distress (CHU9D ≥ 20). All regression models accounted for school-level clustering and incorporated school-level variables (e.g.: total number of students in the school, whether the school also participated in swab testing or not, and the School Index of Community Socio-Educational Advantage (ICSEA), which was used to provide an indication of the school-level socio-educational context). Significance levels were set at *p* < 0.05.

## Results

### Participants

In total, 40 schools with secondary students took part in the data collection. In C1, 19,240 students in Grades 7– 12 participated in data collection (49.0% male, 48.0% female, 2.7% ‘other’ gender) and 13,609 in C2 (49.0% male, 48.0% female, 2.7% ‘other’). Within the student respondent cohort, a greater proportion of LOTE students (C1: 28.5%, C2: 26.5%) responded compared to the broader Western Australian public school student population (WA: 20.5%), however Aboriginal and Torres Strait Islander students (C1: 5.8%, C2: 6.4%) were under-represented in the study (WA: 8.5%). See Table 1 for further demographic details.

**Table 1:** Demographics of secondary student participants

	C1		C2	
	n	%	n	%
Grade level				
Grade 7	3949	16.5	3304	18.3
Grade 8	3598	15.0	2986	16.6
Grade 9	3438	14.3	3030	16.8
Grade 10	3417	14.2	2397	13.3
Grade 11	2842	11.8	1609	8.9
Grade 12	2012	8.4	342	1.9
Gender				
Male	9482	49.0	6691	49.0
Female	9242	48.0	6552	48.0
Other	516	2.7	366	2.7
Language Other Than English (LOTE)				
Yes	5471	28.5	3599	26.5
No	13,691	71.5	9968	72.3
Aboriginal and/or Torres Strait Islander				
Yes	1113	5.8	870	6.4
No	18,013	94.2	12,667	93.6
Condition				
Survey only	9639	50.1	7012	51.5
Swab Testing & survey	9602	49.9	6603	48.5
Residential College				
Yes	104	0.5	76	0.6
No	19,150	99.5	13,564	99.4
Educational Region				
Goldfields	486	2.5	384	2.8
Kimberley	445	2.3	339	2.5
Midwest	58	0.3	55	0.4
North Metropolitan	8447	43.9	4825	35.4
Pilbara	231	1.2	253	1.9
South Metropolitan	7521	39.1	5994	44.4
Southwest	1755	9.1	1565	11.5
Wheatbelt	311	1.6	225	1.6

### Descriptive data for the Child Health Utility Index (CHU9D)

**Fig. 2:**
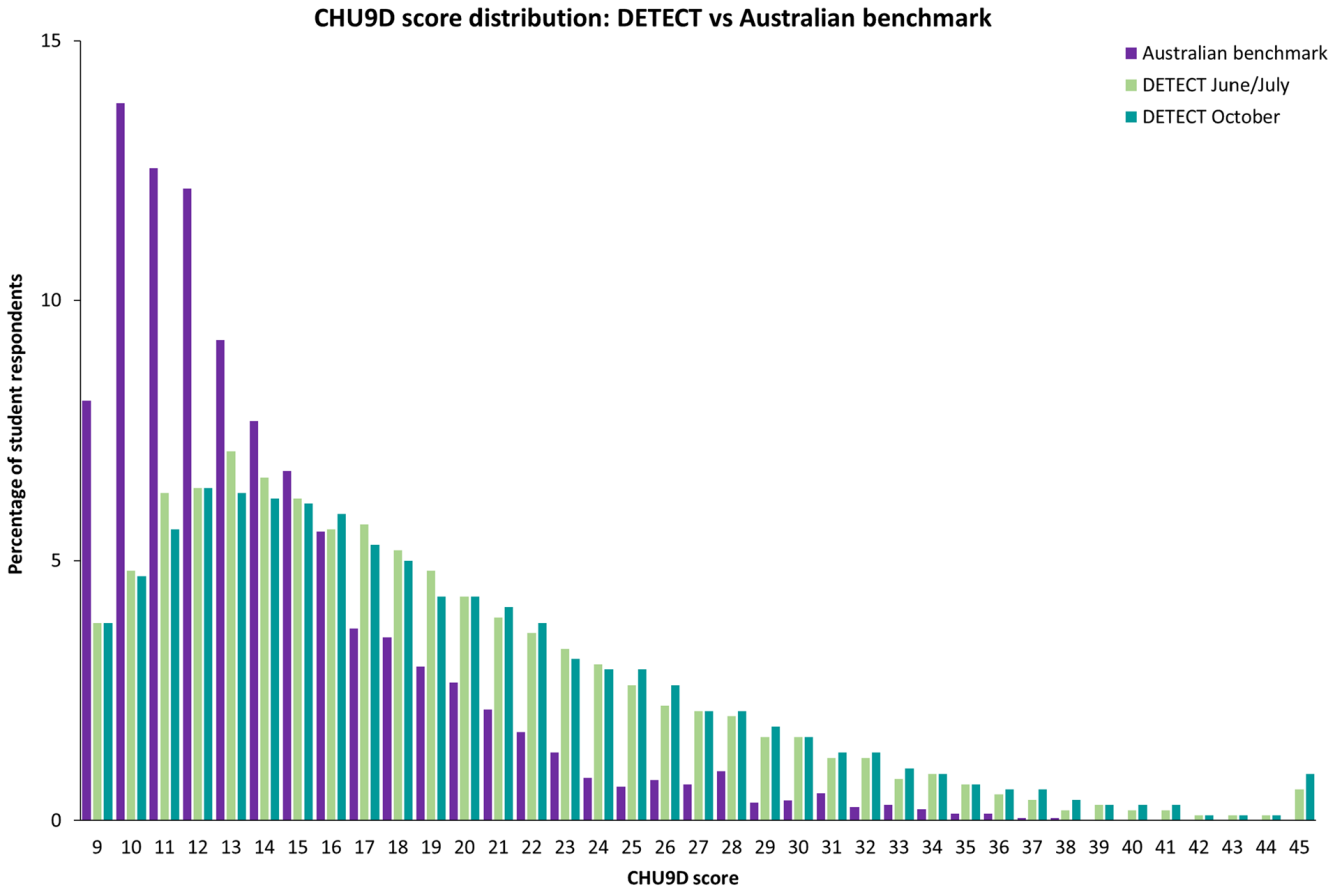
Comparative distribution of summed CHU9D scores for DETECT Western Australian secondary student respondents (Cycles 1 and 2) and Australian benchmark data from the Young Minds Matter survey (2013/2014)

Scores of 20–25 on the CHU9D were established as a threshold for moderate difficulties and distress, and scores over 25 for high levels. The benchmark data from 2013/2014 indicated that 14.4% of students scored ≥ 20 on the CHU9D. By comparison, 36% of adolescents at C1 and 39% at C2 scored 20 or above; 18% of students were classified as reporting moderate difficulties (CHU9D score of 20–25) in both Cycles 1 and 2. At C1, 18% of participants reported high levels of difficulties, and by C2, this increased to 21% of respondents. Most students reported CHU9D scores below these thresholds (C1: 63%; C2: 61%; see Table 2 for percent of students in each range by school Grade and gender). Figure 2 provides a graphic representation of the difference in score distributions from 2014 compared to the two cycles of data collection in 2020 during COVID-19. This demonstrates the magnitude of difference in difficulties and emotional distress reported by WA students sampled during the COVID-19 pandemic compared with in 2013/2014.

**Table 2:** Percentage of students scoring below CHU9D thresholds, and at moderate and high range, by grade, gender, and survey cycle

%		C1			C2		
		Not elevated	Moderate	High	Not elevated	Moderate	High
Grade 7	Male	76.7	14.3	8.9	74.9	14.4	10.7
	Female	65.9	16.3	17.8	58.0	19.5	22.5
	Total	71.5	15.3	13.2	66.6	16.9	16.5
Grade 8	Male	75.4	13.5	11.1	72.4	15.5	12.1
	Female	57.7	20.2	22.2	52.4	19.7	27.9
	Total	66.4	16.9	16.7	62.3	17.6	20.1
Grade 9	Male	70.6	15.7	13.7	69.8	14.7	15.6
	Female	50.3	23.2	26.5	48.7	22.5	28.8
	Total	60.4	19.5	20.2	59.0	18.7	22.3
Grade 10	Male	70.3	15.5	14.2	67.8	15.7	16.5
	Female	52.1	21.0	26.9	45.2	22.3	32.6
	Total	61.3	18.2	20.5	56.4	19.0	24.6
Grade 11	Male	69.3	15.0	15.6	60.9	19.4	19.7
	Female	51.4	23.3	25.3	49.4	21.7	29.0
	Total	60.5	19.1	20.4	55.3	20.5	24.1
Grade 12	Male	65.0	19.1	15.9	72.5	12.3	15.2
	Female	47.2	25.1	27.7	53.1	19.7	27.2
	Total	55.5	22.3	22.2	62.5	16.1	21.4
Total	Male	71.9	15.2	12.8	70.2	15.5	14.3
	Female	54.9	21.1	24.0	51.3	21.0	27.8
	Total	63.4	18.2	18.4	60.7	18.2	21.0

On average, secondary students reported significantly higher CHU9D scores at C2 (M = 19.1, SD = 7.6) than at C1 (M = 18.6, SD = 7.2) (*F* = 38.8, df = 31,201, *p* < 0.001). At both time points, difficulties and distress increased with age (F = 66.78, *p* < 0.001), and females (M = 20.1, SD = 7.3) reported significantly higher CHU9D scores than males (M = 17.1, SD = 6.6) (*F* = 1368.964, *p* < 0.001).

The CHU9D items that presented the most common problems for students were feeling tired (C1: M = 3.2, C2: M = 3.3), followed by feeling annoyed (C1: M = 2.1, C2: M = 2.2), having problems with sleep (C1: M = 2.1, C2: M = 2.2), and having problems with schoolwork (C1: M = 2.0, C2: M = 2.0). Students also reported not being able to join in with activities (C1: M = 1.9, C2: M = 2.0), feeling sad (C1: M = 1.9, C2: M = 2.0), feeling worried (C1: M = 1.9, C2: M = 1.9), feeling pain (C1: M = 1.9; C2: M = 2.0), and problems with daily routine (C1: M = 1.6, C2: M = 1.7). See Additional file 1: Tables S1 and S2 for C1 and C2 item breakdowns by gender and grade level.

### Predictors of moderate-high difficulties and distress

In both C1 and C2, females were significantly more likely to report elevated difficulties and distress than males (C1 OR = 2.3, *p* < 0.001; C2 OR = 2.3, *p* < 0.001). At C1, students in higher grades (Grade 9–12) were more likely to report elevated difficulties and distress (OR ranges from 1.2 to 1.7, *p* < 0.001); at C2, only students in Grades 10 and 11 reported significantly greater distress than the younger students (see Table 3), although there were many fewer Grade 12 students reporting at C2 (342, down from 2,012 at C1). The proportion of students categorised as exhibiting elevated distress significantly increased by Grade level for males (C1: χ^2^ = 59.539, *p* < 0.01; C2: χ^2^ = 54.486, *p* < 0.01) and females (C1: χ^2^ = 138.6, *p* < 0.01; C2: χ^2^ = 49.0, *p* < 0.01). A significantly higher proportion of females than males reported elevated distress at all Grade levels (C1 & C2: all *p* < 0.01).

**Table 3:** Logistic regression results for elevated (moderate & high) levels of difficulties and distress for C1 and 2 data collections

	C1				C2			
	OR	LCI	UCI	p	OR	LCI	UCI	p
Individual-level predictors								
Gender (male)	2.34	2.18	2.52	< 0.001**	2.31	2.12	2.52	< 0.001**
Grade level								
Grade 8	1.06	0.94	1.19	0.365	1.01	0.88	1.15	0.872
Grade 9	1.21	1.07	1.36	0.002**	1.07	0.95	1.22	0.273
Grade 10	1.24	1.10	1.39	0.004**	1.27	1.11	1.44	< 0.001**
Grade 11	1.27	1.12	1.44	< 0.001**	1.48	1.27	1.66	< 0.001**
Grade 12	1.72	1.50	2.00	< 0.001**	1.23	0.92	1.66	0.166
LOTE	1.00	0.92	1.09	0.940	0.96	0.92	1.06	0.417
Aboriginal and/or Torres Strait Islander	0.94	0.79	1.11	0.489	0.85	0.70	1.03	0.102
Educational region								
Goldfields	0.88	0.60	1.30	0.527	0.80	0.59	1.08	0.139
Kimberley	0.56	0.39	0.82	0.002**	0.75	0.55	1.03	0.079
Midwest	0.83	0.35	1.99	0.684	0.53	0.23	1.21	0.132
North Metropolitan	1.03	0.89	1.18	0.695	1.11	1.01	1.23	0.035*
Pilbara	0.65	0.42	0.99	0.050	1.03	0.73	1.45	0.875
Southwest	0.91	0.73	1.13	0.420	0.74	0.63	0.88	0.001*
Wheatbelt	1.09	0.73	1.63	0.670	0.85	0.58	1.23	0.385
#people in home	0.98	0.95	1.00	0.137	0.98	0.95	1.02	0.297
Number of friends	0.89	0.87	0.92	< 0.001**	0.89	0.87	0.92	< 0.001**
Quality of friends	0.70	0.67	0.73	< 0.001**	0.73	0.69	0.77	< 0.001**
School connectedness	0.83	0.82	0.83	< 0.001**	0.82	0.81	0.83	< 0.001**
School-level predictors								
ICSEA	0.99	0.95	1.01	0.400	1.00	1.00	1.00	0.037*
Number of students in school	1.00	1.00	1.00	0.118	1.00	1.00	1.00	0.223
Study arm	0.96	0.84	1.10	0.573	1.06	0.95	1.17	0.263

**p < 0.01. Reference groups - Distress: not distressed; Gender: male; Grade level: Grade 7; LOTE: No; Aboriginal and/or Torres Strait Islander: No; Region: South metropolitan; Study arm: swab testing

Potential protective factors were observed. Students who reported a higher number of friends (OR = 0.90 for C1 and C2, *p* < 0.001) and those who reported greater quality of friendships (OR = 0.7 for C1 and C2, *p* < 0.001) were at reduced risk of elevated difficulties and distress. In addition, students who reported higher school connectedness (OR = 0.8 for C1 and C2, *p* < 0.001) were also less likely to report elevated difficulties and distress.

## Discussion

Among the many concerns arising from the COVID-19 pandemic is the risk that such an unprecedented global event might affect the wellbeing of children and young people. We report higher levels of difficulty and distress during the pandemic in Western Australian adolescents than previously documented, even though rates of COVID-19 remained low in Western Australia throughout 2020. Although the study design does not permit a causal conclusion, the increase—from 14.4% of respondents scoring above the threshold in 2013/2014 to 39% by October 2020 – is well beyond the standard error of prevalence estimates typically produced in epidemiological studies of populations. This magnitude of change is in stark contrast with reports of little change in the estimated prevalence of more severe mental disorders in Australian 6–17 year-olds between 1998 and 2014 [30].

As the last dataset for comparison was collected six years prior, the marked increase in difficulties and distress in 2020 could reflect a broader societal shift in adolescent wellbeing not entirely due to the COVID-19 pandemic. For example, other Western Australian data show a 50% increase from 2015 to 2019 in the number of children aged 0–17 years referred to the Western Australia Child and Adolescent Mental Health Service [31]. The Mission Australia Youth Survey has found growing rates of emotional distress, from 18.6% of their respondents in 2012 to 27.0% in 2019, prior to the pandemic, with comparable gender differences indicating girls are more distressed than boys, as was found here [13]. However, in light of post-COVID-19 studies from other countries [6–8] and findings such as a 104% increase during 2020 in Western Australian children with anorexia nervosa requiring admission to the hospital compared to the previous three years [32], it seems plausible that the global pandemic has played a role in the 2.7-fold increase in prevalence of adolescent difficulties and distress found in this study.

The possibility that the pandemic played a role in this marked difference in wellbeing is further supported by the significant increase in difficulties and distress at Cycle 2, collected 7–8 months after the first COVID-19 cases in Western Australia. This might indicate that the ongoing unresolved global nature of the pandemic was still exerting an influence on adolescent wellbeing. Although causal inference cannot be drawn due to the design of the study, there are research precedents to support interpretation that the events associated with COVID-19, even in a setting such as Western Australia with relatively few cases, could reasonably have contributed to the elevated adolescent difficulties and distress. For example, research arising after the 9/11 terrorist attacks illustrated that such historical events may affect the mental health and wellbeing of young people who were not directly affected by the attack [33]. Experimental psychologists have found that tasks that entail reflecting on existential threats can increase negative affect [34], and from a stress and coping perspective (e.g., [35]) a global pandemic constitutes an uncontrollable and dangerous stressor providing no recourse for problem-focused coping. Exposure to media reports and awareness of the global and uncontrollable nature of the pandemic may be sufficient to imperil wellbeing.

Another possible reason for the increase in difficulties and distress since the 2013/2014 benchmark may be a shift in willingness to talk about one’s emotional struggles. Australia has seen a notable increase in public health messaging regarding mental health; for example, the *R U OK* initiative [36] has increased awareness of this issue. Following an unprecedented decade of de-stigmatisation and promotion of mental health and seeking mental health care, the pandemic may have catalysed a further broad discussion of mental health, potentially ameliorating some of the stigma associated with speaking openly about emotional wellbeing and thereby increasing the likelihood that a young person feels comfortable divulging if they are experiencing difficulties.

The findings reveal that about 39% of students are reporting at least moderate to high levels of difficulties and emotional distress. They reported being tired, having trouble sleeping and being annoyed, and higher proportions of them also said they were sad and worried. While the study is not designed to test whether the pandemic is responsible, or the degree to which it is, these difficulties have a high valence against the circumstances confronting young people and the uncertainty of future outcomes.

Although this study can neither definitively attribute the increased difficulties and distress to the pandemic, nor draw conclusions about resilience in the face of the crisis, it did examine predictors of difficulties. Gender and grade level were both associated with wellbeing outcomes, with female students and those in older Grade levels more likely to present with elevated difficulties and distress than their counterparts. These associations observed during the pandemic are consistent with wellbeing predictors reported in 2013/2014 [37]. Older students were contending with exit examinations, university entrance and significant social events such as school formals being interrupted or cancelled at the time of the study. Further to this, greater capacities for abstract cognition may enable older students to be more conscious of the potential for a global pandemic to adversely affect their lives; while younger students may be more circumscribed about the impact of the pandemic on their lives, and more influenced by concrete aspects of their lives, such as being permitted to return to ‘normal’ schooling. The quality and quantity of friendships and a sense of connectedness with the school community were consistently associated with lower risk of difficulties and distress, as has been established in past research for more acute mental health and wellbeing risk factors [19, 38].

### Limitations

As noted, we cannot conclude that the elevated rates of difficulty and distress observed were caused by the COVID-19 pandemic. As well, in considering these data, it is important to remember that the CHU9D reflects students’ difficulties and distress in general. As this measure is based on students’ reported experiences “today”, the results may reflect relatively transient states. In addition, while previous research has demonstrated that the CHU9D is an appropriate and valid routine outcome measure of adolescent mental health [39], it is not a clinical diagnostic tool, so students with elevated scores are not necessarily experiencing mental illness or syndromes, although they are at greater risk than their peers of doing so.

Students from private, non-government schools are not represented here, and the data presented here are not indicative of the wellbeing of all young people in Western Australia. In Australia, private non-government schools comprise the Independent Schools’ sector and the Catholic Education Schools’ sector. The independent schools and to a lesser extent the Catholic schools sectors have larger proportions of students from high socioeconomic backgrounds and smaller proportions of students from low socioeconomic backgrounds, compared to government public school [40]. More regular collection of these data will be critical to identify which areas, schools or students are at highest risk for emotional difficulties moving forwards, and to monitor the longitudinal impact of the pandemic on wellbeing in the coming years.

As the wellbeing measure (the CHU9D) has not been validated for use with Aboriginal and Torres Strait Islanders children and adolescents, results for this population should be treated with caution. Other Australian studies have found elevated emotional distress among Aboriginal and Torres Strait Islanders young people [13], so the non-significant difference observed here is unexpected and may be due to inadequacies of the instrument in addressing wellbeing and quality of life amongst this population.

The study experienced high rates of attrition amongst the Grade 12 students (C1:2,012; C2:342). This is likely due to the timing of the study, with the 2^nd^ data collection occurring in late October/early November, when graduating students are preparing for and/or sitting their final examinations. This attrition requires results to be treated with caution for these older students.

## Conclusions

Our data—collected rapidly from a large and broadly representative cohort of public-school students at a time when insight into how children and young people were tracking was critical—provide insight into the current state of adolescent wellbeing in a low prevalence setting where some of the most obvious negative impacts of COVID-19 (i.e., high local infection or death rates; limitations to personal freedoms for long periods) were not directly present in the local community. The picture is not good. Whether due to the pandemic, to other secular changes, or – most likely – to the convergence of the two, these findings represent a major public health concern, that warrants an urgent response.

Our findings were only made possible through the use of passive/opt-out parental consent [41] and demonstrate that this form of data collection is feasible even in a time of significant resource scarcity and competing priorities. These findings highlight the need for more robust systematic monitoring of adolescent wellbeing and mental health to track changes over time, and to provide a benchmark against which future studies and interventions can be evaluated; including the evidence-based community capacity building and intervention implementation required to address the significant wellbeing needs of communities during this challenging new era.

This knowledge could be utilised to deliver equity by allocating limited resources more precisely to support the types of prevention and response that may be the most appropriate based on students’ needs, and to assess the effectiveness and return on investment of these policies and practices over time. Regional inequities observed in our data are an ongoing challenge for Australia, a very large continent in area and a relatively small population, leading to extreme remote conditions unfound in most nations of the world. For many regional communities, lower levels of health service use result in reduced access to service [42]. This tyranny of distance disproportionately affects Australia’s first peoples, who make up a larger proportion of Australia’s regional and remote populations, compared to urban areas [43]. Schools provide a hub for engaging regional and remote families, and consultation and active engagement with Aboriginal Elders and other key stakeholders toward in-depth research to support regional and remote students is needed.

System-level wellbeing prevalence data could also inform and support whole-school wellbeing planning, staff training and resource allocation for tailored, school-level evidence-based prevention, early detection and treatment policies and practices. Ultimately, this will guide government resource prioritisation, decision making and action to reduce the high rates of mental health problems in our communities.

## Supplementary Information

**Additional file 1: Table S1.** CHU9D item mean scores and total CHU9D scores by grade, gender, and survey cycle at Cycle 1 (June 2020). **Table S2.** CHU9D item mean scores and total CHU9D scores by grade, gender, and survey cycle at Cycle 2 (October 2020).

## Data Availability

The datasets used and/or analysed during the current study are available from the corresponding author on reasonable request.

## Abbreviations

C1: Cycle 1
C2: Cycle 2
CHU9D: Child Health Utility Index
COVID-19: Coronavirus 19
ICSEA: School Index of Community Socio-Educational Advantage
K10: Kessler 10
LOTE: Language other than English
OR: Odds ratio
WA: Western Australia(n)

## Footnotes

1 Analysis available upon reasonable request.
