# Supplementary material for: Western Australian adolescent emotional wellbeing during the COVID-19 pandemic in 2020
Source: Child Adolesc Psychiatry Ment Health. 2022 Jan 13;16:4. doi: 10.1186/s13034-021-00433-y (PMC8756750; doi:10.1186/s13034-021-00433-y)
Supplement: Supplementary file 1 — Additional file 1: Table S1. CHU9D item mean scores and total CHU9D scores by grade, gender, and survey cycle at Cycle 1 (June 2020). Table S2. CHU9D item mean scores and total CHU9D scores by grade, gender, and survey cycle at Cycle 2 (October 2020). [file 13034_2021_433_MOESM1_ESM.docx]

Additional file 1: Table S1. CHU9D item mean scores and total CHU9D scores by grade, gender, and survey cycle at Cycle 1 (June 2020).

| Grade | Gender | Feel worried | Feel sad | Feel pain | Feel tired | Feel annoyed | Problems with school work | Problems with sleep | Problems with routine | Problems with daily activities | CHU9D Total |
| --- | --- | --- | --- | --- | --- | --- | --- | --- | --- | --- | --- |
| Grade 7 | Male | 1.5 | 1.5 | 1.7 | 2.8 | 1.8 | 1.8 | 1.9 | 1.4 | 1.7 | 16.1 |
|  | Female | 1.8 | 1.9 | 1.9 | 3.0 | 2.1 | 1.8 | 2.1 | 1.5 | 1.7 | 18.0 |
|  | Total | 1.7 | 1.7 | 1.8 | 2.9 | 1.9 | 1.8 | 2.0 | 1.5 | 1.7 | 17.0 |
| Grade 8 | Male | 1.6 | 1.6 | 1.7 | 2.9 | 1.9 | 1.9 | 1.9 | 1.4 | 1.7 | 16.6 |
|  | Female | 2.0 | 2.1 | 2.0 | 3.3 | 2.3 | 2.0 | 2.3 | 1.6 | 1.9 | 19.4 |
|  | Total | 1.8 | 1.8 | 1.8 | 3.1 | 2.1 | 2.0 | 2.1 | 1.5 | 1.8 | 18.0 |
| Grade 9 | Male | 1.6 | 1.6 | 1.8 | 3.0 | 2.0 | 2.0 | 1.9 | 1.5 | 1.8 | 17.1 |
|  | Female | 2.1 | 2.2 | 2.0 | 3.5 | 2.4 | 2.1 | 2.3 | 1.7 | 2.0 | 20.3 |
|  | Total | 1.9 | 1.9 | 1.9 | 3.2 | 2.2 | 2.0 | 2.1 | 1.6 | 1.9 | 18.7 |
| Grade 10 | Male | 1.7 | 1.6 | 1.8 | 3.0 | 2.0 | 2.0 | 1.9 | 1.5 | 1.8 | 17.3 |
|  | Female | 2.1 | 2.2 | 2.0 | 3.5 | 2.3 | 2.1 | 2.3 | 1.7 | 2.0 | 20.4 |
|  | Total | 1.9 | 1.9 | 1.9 | 3.2 | 2.2 | 2.0 | 2.1 | 1.6 | 1.9 | 18.8 |
| Grade 11 | Male | 1.7 | 1.7 | 1.7 | 3.0 | 2.0 | 2.0 | 2.1 | 1.5 | 1.9 | 17.6 |
|  | Female | 2.1 | 2.2 | 1.9 | 3.6 | 2.3 | 2.0 | 2.2 | 1.7 | 2.1 | 20.3 |
|  | Total | 1.9 | 2.0 | 1.8 | 3.3 | 2.2 | 2.0 | 2.1 | 1.6 | 2.0 | 18.9 |
| Grade 12 | Male | 1.9 | 1.8 | 1.8 | 3.1 | 2.0 | 2.0 | 2.0 | 1.6 | 1.9 | 17.9 |
|  | Female | 2.3 | 2.2 | 1.9 | 3.7 | 2.3 | 2.1 | 2.4 | 1.7 | 2.2 | 20.8 |
|  | Total | 2.1 | 2.0 | 1.8 | 3.4 | 2.2 | 2.0 | 2.2 | 1.6 | 2.0 | 19.4 |
| Total | Male | 1.6 | 1.6 | 1.7 | 2.9 | 1.9 | 1.9 | 2.0 | 1.5 | 1.8 | 17.0 |
|  | Female | 2.1 | 2.1 | 1.9 | 3.4 | 2.3 | 2.0 | 2.3 | 1.7 | 2.0 | 19.8 |
|  | Total | 1.9 | 1.9 | 1.8 | 3.2 | 2.1 | 2.0 | 2.1 | 1.6 | 1.9 | 18.4 |
| Note. Range of item scores 1-5; 1=lowest, 5=highest; total score range 9 – 45. 20+ represents threshold for moderate difficulties and distress | | | | | | | | | | | |

Additional file 1: Table S2. CHU9D item mean scores and total CHU9D scores by grade, gender, and survey cycle at Cycle 2 (Oct 2020).

| Grade | Gender | Feel worried | Feel sad | Feel pain | Feel tired | Feel annoyed | Problems with school work | | Problems with sleep | Problems with routine | Problems with daily activities | CHU9D Total |
| --- | --- | --- | --- | --- | --- | --- | --- | --- | --- | --- | --- | --- |
| Grade 7 | Male | 1.5 | 1.5 | 1.8 | 2.9 | 1.8 | | 1.8 | 1.9 | 1.5 | 1.7 | 16.4 |
|  | Female | 1.9 | 2.1 | 2.0 | 3.3 | 2.2 | | 1.9 | 2.3 | 1.6 | 1.9 | 19.2 |
|  | Total | 1.7 | 1.8 | 1.9 | 3.1 | 2.0 | | 1.8 | 2.1 | 1.5 | 1.8 | 17.8 |
| Grade 8 | Male | 1.6 | 1.6 | 1.8 | 3.0 | 2.0 | | 1.9 | 2.0 | 1.5 | 1.8 | 16.9 |
|  | Female | 2.1 | 2.2 | 2.1 | 3.5 | 2.4 | | 2.0 | 2.3 | 1.7 | 2.0 | 20.3 |
|  | Total | 1.8 | 1.9 | 1.9 | 3.2 | 2.2 | | 2.0 | 2.1 | 1.6 | 1.9 | 18.6 |
| Grade 9 | Male | 1.7 | 1.7 | 1.9 | 3.0 | 2.1 | | 1.9 | 2.0 | 1.5 | 1.8 | 17.4 |
|  | Female | 2.2 | 2.3 | 2.1 | 3.6 | 2.5 | | 2.1 | 2.3 | 1.7 | 2.1 | 20.9 |
|  | Total | 1.9 | 2.0 | 2.0 | 3.3 | 2.3 | | 2.0 | 2.2 | 1.6 | 1.9 | 19.2 |
| Grade 10 | Male | 1.7 | 1.8 | 1.8 | 3.0 | 2.0 | | 2.0 | 2.0 | 1.5 | 1.9 | 17.7 |
|  | Female | 2.3 | 2.3 | 2.2 | 3.7 | 2.6 | | 2.2 | 2.4 | 1.9 | 2.2 | 21.6 |
|  | Total | 2.0 | 2.1 | 2.0 | 3.3 | 2.3 | | 2.1 | 2.2 | 1.7 | 2.0 | 19.7 |
| Grade 11 | Male | 1.9 | 1.8 | 1.8 | 3.2 | 2.1 | | 2.1 | 2.1 | 1.6 | 2.0 | 18.7 |
|  | Female | 2.4 | 2.2 | 1.9 | 3.6 | 2.4 | | 2.1 | 2.3 | 1.7 | 2.2 | 20.8 |
|  | Total | 2.1 | 2.0 | 1.9 | 3.4 | 2.2 | | 2.1 | 2.2 | 1.7 | 2.1 | 19.7 |
| Grade 12 | Male | 1.9 | 1.7 | 1.6 | 2.7 | 1.8 | | 1.7 | 2.0 | 1.5 | 1.9 | 16.8 |
|  | Female | 2.3 | 2.3 | 2.0 | 3.5 | 2.2 | | 1.9 | 2.4 | 1.7 | 2.3 | 20.6 |
|  | Total | 2.1 | 2.0 | 1.8 | 3.1 | 2.0 | | 1.8 | 2.2 | 1.6 | 2.1 | 18.7 |
| Total | Male | 1.7 | 1.7 | 1.8 | 3.0 | 2.0 | | 1.9 | 2.0 | 1.5 | 1.8 | 17.3 |
|  | Female | 2.1 | 2.2 | 2.1 | 3.5 | 2.4 | | 2.0 | 2.3 | 1.7 | 2.1 | 20.5 |
|  | Total | 1.9 | 1.9 | 1.9 | 3.2 | 2.2 | | 2.0 | 2.1 | 1.6 | 1.9 | 18.9 |
| Note. Range of item scores 1-5; 1=lowest, 5=highest; total score range 9 – 45. 20+ represents threshold for moderate emotional distress | | | | | | | | | | | | |
